# Fostering Playfulness in 2-to-6-Year-Old Children: A Longitudinal Study of Parental Play Support Profiles and Their Effects on Children’s Playfulness

**DOI:** 10.3390/bs15121716

**Published:** 2025-12-11

**Authors:** Isabelle Duss, Cornelia Rüdisüli, Corina Wustmann Seiler, Patricia Lannen

**Affiliations:** 1 Institute of Education, University of Zurich, 8032 Zurich, Switzerland; 2 Marie Meierhofer Children’s Institute (MMI), Associated Institute of the University of Zurich, 8005 Zurich, Switzerland; lannen@mmi.ch; 3 Schaffhausen University of Teacher Education (PH Schaffhausen), 8200 Schaffhausen, Switzerland; cornelia.ruedisueli@phsh.ch; 4 Department of Research and Development, Zurich University of Teacher Education (PH Zurich), 8090 Zurich, Switzerland; corina.wustmann@phzh.ch

**Keywords:** parental play support, children’s playfulness, latent profile analysis, latent profile transition analysis, longitudinal study

## Abstract

Parental play support is crucial to children’s playfulness. Using a person-centered approach, we identified profiles of parental play support based on self-assessed roles. In total, 447 mothers and fathers reported their roles—co-player, playleader, director, uninvolved, or onlooker—during their child’s play. These roles were assessed at two time points, spaced two years apart. Latent profile analysis revealed three distinct profiles: (1) engaged play support, (2) versatile play support, and (3) non-interfering play support. Parental affiliation to one of the three profiles was very stable over time, with only 14% of parents transitioning between profiles. Our longitudinal analysis indicated that the engaged play support profile, in which parents are closely involved in their child’s play either by playing with the child or observing children’s play, was significantly related to children’s playfulness two years later. Notably, 28.4% of parents fell into this profile. These findings underscore the importance of active parental engagement in fostering children’s playfulness.

## 1. Introduction

Play is the daily business of children in their early years and is linked to healthy physical and cognitive development (Bento & Dias, 2017; Lai et al., 2018; Whitebread et al., 2017). The family is assumed to be the primary environment for children’s development (Bronfenbrenner, 1979) and parents have been found to have a central role in children’s play (Jensen et al., 2021; LaForett & Mendez, 2017). Consequently, parents can foster their children’s play (LaForett & Mendez, 2017). Through parents’ active engagement in children’s play, children can explore the world together with their parents. At the same time, parents can observe how their children experience their own world (Ginsburg, 2007). Furthermore, by actively engaging in their children’s play, parents can act as role models for learning and thus foster their children’s development of various skills (Bandura, 1977). However, the way in which parents provide this play support can vary significantly, and there is little empirical consensus on what effective play support should look like (Trawick-Smith & Dziurgot, 2011).

### 1.1. Parental Play Support

Most parents have a positive attitude towards the central role of play in their children’s development and are willing to actively support their children’s play (Parmar et al., 2008). However, it remains empirically unclear with which intensity and intention the most effective play support takes place (Skene et al., 2022). Some experts (Frost et al., 2005; McInnes et al., 2011) advise that children should be left to organize their own play without any adult involvement, because too much adult involvement can lead to children gaining less experience. Children can perceive adults’ involvement as intrusive, leading to a loss of interest and a disruption in the play activity (Gmitrová & Gmitrov, 2003; Weldemariam, 2014). When children play on their own, they develop decision-making abilities and uncover personal interests (Moreno, 2016), and they can become autonomous (Veiga et al., 2016). Conversely, other experts assert that the direct involvement of adults in children’s play is necessary. Through direct involvement, adults can promote specific play skills, which in turn have a positive effect on aspects of children’s development, such as their social development (Singer et al., 2014; Prada-Mateus et al., 2024). Zosh et al. (2017) noted that both free, child-centered play and adult-guided play are important for children’s development and thus that both perspectives on children’s play should be brought together. Adults should adapt their play support to children’s current needs and development (Perren et al., 2019; Trawick-Smith & Dziurgot, 2011).

Studies have shown that parental play support is influenced by various factors, such as the children’s gender, the presence of siblings, and parental characteristics. A child’s gender is a predictor of parental play support: Both mothers and fathers tend to engage more in play activities with their daughters than with their sons (Elkind, 2010; John et al., 2013). Additionally, in families with multiple children, parents tend to be less engaged in play than in families with only one child (Wustmann Seiler et al., 2021). Beyond child-related factors, parental age has also been linked to parenting behaviors: older maternal age has been found to be slightly negatively associated with mothers’ self-reported playfulness (Tandler & Proyer, 2022), which may in turn affect their engagement in play activities with their children.

### 1.2. Change in Parental Play Support During Children’s Play

During play with their children, parents can tailor their support to children’s developmental and contextual needs. For example, parents apply more direct guidance and control during play when children are younger and less cognitively able (Robinson et al., 2021). As children grow, parents perceive their involvement in play as less essential, which may be explained by a shift in focus from play to academic skills upon kindergarten entry (Warash et al., 2017). Similarly, in Wustmann Seiler et al.’s (2021) study with children between 2 and 8 years, parents were more involved in children’s play when their child was younger. Notably, no explicit findings are available on stability or changes in parental play support throughout childhood because, to the best of our knowledge, no longitudinal studies have been conducted on this topic yet.

### 1.3. Effects of Parental Play Support on Children’s Playfulness

Research indicates that parental play support significantly affects children’s play (Tamis-LeMonda et al., 2004) and therefore shapes their playfulness: the quality of children’s play (Barnett, 1991; Fung & Chung, 2025). Given the importance of playfulness to children’s well-being (Sanders et al., 2022) and its association with creativity and imagination (Lieberman, 1977), playfulness is a crucial aspect of children’s play (Skard & Bundy, 2011). Lieberman’s (1977) foundational study identified five key dimensions that are central to children’s playfulness: physical spontaneity, cognitive spontaneity, social spontaneity, manifest joy, and sense of humor. These five dimensions collectively indicate the comprehensive nature of children’s play behavior. Theoretical insights from Youell (2008) suggest that a child’s playfulness develops through interactions with adults. For instance, parental playtime with a child and parental behaviors that encourage a child’s engagement in play correlate positively with children’s playfulness (Duss et al., 2024; Fung & Chung, 2022; Waldman-Levi, 2021). One way of examining how parents support children’s play is to investigate the roles that parents actually assume during their child’s play (Parmar et al., 2008).

### 1.4. Parental Roles During Children’s Play

Previous interview studies with mothers in various cultural contexts and observations of parent–child play sequences have identified four distinct roles that parents adopt during their child’s play (Muhonen et al., 2019; Russell & Saebel, 1997): (1) Co-players are parents who participate in their child’s play but leave the leadership to the child and do not dominate their play and therefore play at an equal level with their child. (2) Playleaders are described as parents who enhance their child’s play experience by offering suggestions, posing questions, and providing demonstrations. They also offer support to their child when the child encounter difficulties or struggles to engage in play. Parents can fulfill this role either by actively participating in their child’s play or by offering guidance and assistance from outside the play. (3) Directors are parents who provide information and instructions to their child during play, thus exercising control over their child’s play. (4) Onlookers are parents who remain in the background and observe their child’s play and interests. In the study by Russell and Saebel (1997) the role of playleader was observed most frequently, followed by the director role. The role of co-player was observed least frequently. Mothers in a study by Muhonen et al. (2019) also reported that they participated primarily in the roles of playleader and director. They were less likely to adopt the roles of co-player or onlooker. The roles of teachers during children’s play in educational settings have been discussed more extensively, with distinctions being made between more roles (e.g., Ivrendi, 2020; Rüdisüli et al., 2023) than are typically considered relevant in home settings. However, one additional role that can also be relevant in the home setting is that of uninvolved parent. While their child is playing, uninvolved parents are busy with other tasks such as cooking, household chores, office work, and attending to a sibling.

### 1.5. Summary and Research Questions

Parents can support their children’s play in different ways. Some parents are more active and instructive than others (LaForett & Mendez, 2017). How parents effectively support children’s play reflects their belief about the values and purposes of play (Parmar et al., 2008). However, what play support looks like can vary widely. Additionally, parental play support is correlated with children’s playfulness (Fung & Chung, 2022; Waldman-Levi, 2021; Wustmann Seiler et al., 2021). Furthermore, studies have demonstrated that parental play support varies according to the gender (John et al., 2013) and age (Robinson et al., 2021; Warash et al., 2017) of the child and is influenced by whether the child has siblings or not (Wustmann Seiler et al., 2021). Previous studies have predominantly taken a variable-centered approach by examining individual variables, such as how specific parental behaviors during children’s play influence children’s play (Schneider et al., 2022). Recent studies have employed more psychometrically robust methods to identify latent parenting types across different parenting dimensions using a person-centered approach (Lanza & Cooper, 2016; Luo & Qiu, 2024; Sherrard & Tan, 2024) This approach has a long-standing tradition, tracing back to Baumrind’s (1971) seminal work, which utilized cluster analysis to distinguish different parenting styles. However, only a few studies have examined parenting types from a longitudinal perspective (Cheng et al., 2025). A longitudinal approach is important because it allows for the examination of stability and change in parenting profiles over time, providing insights into developmental dynamics that cross-sectional analyses cannot capture. Building on this foundation, this study aims to identify groups of parents who exhibit similar patterns in their play support roles and examining how these patterns evolve over a two-year period. Accordingly, this study addressed the following questions:

1. Which profiles of parental play support can be identified?
2. How does the profile affiliation of parental play support change over a two-year period?
3. How do children’s age and gender and the presence of siblings affect the profile membership?
4. Which parental play support profile fosters children’s playfulness?

We expect to detect distinct profiles of parental play support characterized by varying levels of parental involvement. Because children grow older and become more independent, parents are expected to be less engaged in children’s play two years later (Robinson et al., 2021). We also expect that affiliation to one of the profiles of parental play support is predicted by child’s and parent’s age and gender, and the presence of siblings (John et al., 2013; Warash et al., 2017; Wustmann Seiler et al., 2021; Tandler & Proyer, 2022). Additionally, we believe that different profiles of parental play support will affect children’s playfulness to varying extents.

## 2. Materials and Methods

### 2.1. Procedure

The present study was part of the Playfulness in early childhood: A longitudinal study of individual and contextual determinants (Playful) project. The Ethics Committee of the Faculty of Philosophy at the University of Zurich, Switzerland, reviewed and approved the study (Ethics approval number 20.12.13). The parents were recruited via 34 childcare centers and 47 kindergartens in 12 cantons in the German-speaking part of Switzerland. In Switzerland, childcare centers are privately funded and privately organized, and they are open to children from the age of 3 months until they enter kindergarten at around 4 years old. Kindergartens are part of the public school system in Switzerland and are for children between the ages of four and six years. The parents and the teachers were informed that their support is voluntary, that the data would be anonymized, that these data would be used only for scientific purposes, and that withdrawal from the study was possible at any time without providing any reason. The longitudinal study took place in spring 2021 (T1) and two years later in spring 2023 (T2). The parents completed an online questionnaire via Survalyzer at T1 about demographic characteristics and at T1 and T2 about their roles during children’s play and their child’s playfulness. The teachers completed the online questionnaire about children’s playfulness at T1 and T2.

### 2.2. Participants

In total, the parents of 447 children (90% mothers) provided information on their roles they assume during children’s play at both timepoints. The mothers were on average 38.9 (*SD* = 4.1) and the fathers 40.8 (*SD* = 7.0) years old. Approximately 62% of the mothers and 76% of the fathers held an academic degree from a university, university of applied sciences, or a university of teacher education. The 447 children were on average 58.2 months old (*SD* = 16.9, range 24–83 months) and 53.5% male. Some 89% of children were Swiss nationals, and German or Swiss-German was spoken in 94% of families. A total of 86 (18.3%) children are only children, 262 (55.9%) have one sibling, and 99 (21.1%) have two or more siblings. All information on family and child characteristics can be found in Table 1.

### 2.3. Instruments

*Parental roles during children’s free play*: Parental support during children’s play was assessed using the Teachers Roles in Free Play Scale (TRFP; Ivrendi, 2020), which was originally developed in English and subsequently translated into German using a forward-backward translation procedure (Wustmann Seiler et al., 2023) and adapted for parents (Wustmann Seiler et al., 2021). The TRFP uses 24 items, which are answered on 5-point Likert scales (1 = never, 5 = very often). The items were distributed across five subscales: (1) co-player (e.g., “If my child wants me to play, I participate as an equal playmate”), (2) playleader (e.g., “If my child can’t get into the play, I support them with play suggestions”), (3) director (e.g., “I decide whom my child will play with”), (4) onlooker (e.g., “I observe my child’s play”), and (5) uninvolved (e.g., “While my child is playing, I am occupied with household work”). The five-factor structure was analyzed using confirmatory factor analysis. Due to the poor model fit at T1 (χ^2^ [242] = 613.0, *p* < 0.001, CFI = 0.837, RMSEA = 0.059, SRMR = 0.063), the role of director was excluded, and seven items were eliminated due to their low factor loadings. These adjustments were applied to both T1 and T2. For each of the remaining four roles, three items were included in the final calculations. An overview of the items is provided in Appendix A. The adjustments resulted in a good model fit for both T1 (χ^2^ [48] = 68.07, *p* = 0.030, CFI = 0.982, RMSEA = 0.031, SRMR = 0.038) and T2 (χ^2^ [48] = 74.16, *p* = 0.009, CFI = 0.981, RMSEA = 0.035, SRMR = 0.039). McDonalds Omega was also acceptable to good for all four roles at both measurements (ω_T1_ = 0.68 to 0.81 and ω_T2_ = 0.70 to 0.83). Furthermore, we were able to demonstrate partial scalar measurement invariance (see Appendix B).

*Children’s playfulness*: The children’s playfulness was assessed with the Children’s Playfulness Scale (CPS; Barnett, 1991) in the German version (Wustmann Seiler et al., 2021). The CPS contains 23 items rated on 5-point Likert scales (1 = does not sound at all like the child; 5 = sounds exactly like the child). The 23 items are distributed across the five dimensions defined by Lieberman (1977): (1) physical spontaneity (4 items, e.g., “The child’s movements are generally well-coordinated during play activities”), (2) cognitive spontaneity (4 items, e.g., “The child invents his/her own games to play”), (3) social spontaneity (5 items, e.g., “The child plays cooperatively with other children”), (4) manifest joy (5 items, e.g., “The child expresses enjoyment during play”), and (5) sense of humor (5 items, e.g., “The child enjoys joking with other children”). Both parents and teachers completed the questionnaire on children’s playfulness at both measurement points. The ratings from parents and teachers showed a significant positive correlation at a medium level at T1 (r_T1_ = 0.34) and a significant positive correlation at a low level at T2 (r_T2_ = 0.26). These ratings from parents and teachers were averaged for each item, and subsequently, the means of the five subscales were computed for each child. The manifest playfulness total score was then derived from these subscales’ mean values. The total playfulness score showed good internal consistency (α_T1/T2_ = 0.83/0.80) and a positive significant test–retest reliability on a medium level (r = 0.44, *p* < 0.001). We evaluated children’s playfulness through reports from both parents and teachers because we recognize that children’s playfulness may vary depending on the situation, environment, and play partners and that parents and teachers may have different perspectives on children’s play activities. By averaging the ratings provided by parents and teachers, a more comprehensive assessment of each child’s overall playfulness can be obtained, which enhances the validity of the measurement and minimizes potential biases from either parent or teacher (Rentzou, 2013).

*Demographic characteristics*: The demographic characteristics collected were child’s age and gender, family nationality and language, parent’s education degree, parent’s age and number of children in the family at T1.

### 2.4. Data Analysis

We performed the latent profile analyses (LPAs) and latent profile transition analysis (LPTA) with Mplus Version 8 (Muthén & Muthén, 2017) and all further analyses with R (R Core Team, 2022). LPA is a statistical procedure that is used to identify latent groups in a sample. Assignment to a group follows a common pattern of responses from several variables (Collins & Lanza, 2010). The LPA takes into account the probability that a person belongs to a certain group and models the uncertainty in the assignment to the group (Spurk et al., 2020). Three steps were taken to observe possible changes in the profiles over two years. First, the LPAs were performed separately for both T1 and T2 to determine the best number of profiles. Second, the latent profiles were named. Third, the LPTA was then calculated with the number of profiles determined in the first step. In the first step, we considered each model cross-sectionally and proceeded in a step-by-step procedure to determine the number of latent profiles. We started with an LPA with one profile and successively added profiles (Nylund et al., 2007). To determine the number of latent profiles, various fit indices were used to select the best model. For the Akaike information criterion (AIC; Akaike, 1974), the Bayesian information criterion (BIC; Schwarz, 1978), and the sample-size adjusted BIC (SABIC; Sclove, 1987), the model with the lowest value fits best in each case. In the Lo–Mendell–Rubin Adjusted Likelihood Ratio Test (ALMR LR; Lo et al., 2001) and bootstrap likelihood ratio test (BLRT; McLachlan & Peel, 2000), a nonsignificant value (*p* < 0.05) suggests that adding an additional profile (k + 1) does not improve the model compared to the current model with k profiles. In this case, the model with k profiles should be retained. For Entropy (Celeux & Soromenho, 1996), a higher value up to the perfect classification of a value 1 indicates a better fit. Clark and Muthén (2009) report a cut-off value of 0.80. However, cut-off values between 0.60 and 0.80 are also considered appropriate (Jung & Wickrama, 2008). The classification accuracy indicates how high the probability is that the case was assigned to the correct profile. No official cut-off value has been defined (Spurk et al., 2020). The maximum likelihood with robust standard errors was used as an estimator to calculate the LPAs. No data were missing for variables relevant to assessing the profiles, and thus, no missing data treatment was required. To guard against local solutions, we increased the default settings in Mplus in line with Hipp and Bauer (2006) and used 7000 random sets of start values, 300 iterations for each random start, and up to 200 final-stage optimizations. In the second step, we visualized the profiles with spider diagrams and named the profiles resulting from the first step. Finally, we calculated the LPTA in the third step. This enabled the identification of changes in parental play support profiles from T1 to T2.

To answer the final question of whether the profiles of parental play support at T1 relate to children’s playfulness at T2, a linear regression was calculated in R. Children’s playfulness at T1 was included as a predictor to control for any initial levels of playfulness when assessing playfulness at T2. Additionally, children’s age and gender were included as control variables due to their predictive significance in previous studies (Barnett, 1991; Keles & Yurt, 2017; Wustmann Seiler et al., 2024).

## 3. Results

### 3.1. Descriptive Statistics and Intercorrelations

The mean values and correlations between the individual roles at T1 and T2 and all other relevant study variables are presented in Table 2. The role of co-player exhibited the highest mean values at T1, whereas the role of uninvolved displayed the highest mean values at T2. Conversely, the role of playleader had the lowest mean values at both time points. Children’s age correlated significantly at a low level with all roles at both measurement points: negatively with the co-player, playleader, and onlooker roles and positively with the uninvolved role. However, gender did not correlate significantly with any of the four roles at either time point. Children’s playfulness at T1 was significantly negatively correlated with the role of playleader at T1 and significantly positively with the uninvolved role at T1, both at a low level. Children’s playfulness at T2 was significantly positively correlated with the onlooker role at both measurement points and with co-player at T2, all at a low level. The presence of siblings correlated significantly positively at a low level with the uninvolved role at both T1 and T2 and significantly negatively at a low level with the co-player and onlooker role at both T1 and T2.

### 3.2. Profiles of Parental Play Support at T1 and T2

A cross-sectional LPA was performed for the four roles at both measurement time points to determine the optimal number of latent profiles. Table 3 presents the fit indices for models ranging from one to six latent profiles for both measurement points. No more profiles were calculated because no more improvements were achieved in the fit indices. Notably, the fit indices consistently supported a solution with three profiles for both measurement points, because when using four profiles, the ALMR LR test was no longer significant, and the number of mothers and fathers in individual profiles were too small to provide reliable conclusions.

To assess whether statistically significant differences occurred in individual roles across the three profiles, a single-factor ANOVA was performed using the mean values of the roles at T1. Because the three profiles contained different sample sizes, a Welch ANOVA was calculated (Eid et al., 2017). As indicated in Table 4, significant differences were observed in all four roles among the three profiles. A post hoc test with Games–Howell correction was used to ascertain the specific differences between profiles. Notably, only the role of playleader did not differ significantly between the second and third profile of parental play support. All other comparisons yielded statistically significant differences.

**Figure 1:**
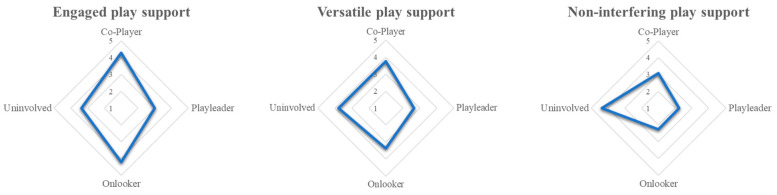
Latent profiles of parental play support at T1.

Next, we described and named the three profiles. The names were chosen to reflect both the nature of parental play support and how the children experience that support during play. To illustrate this visually, the three latent profiles are represented as spider diagrams with their mean values for each role at T1 (see Figure 1). The first latent profile was characterized by high scores for both co-player and onlooker roles and low scores in playleader and uninvolved roles. Parents in this profile were actively engaged in their children’s play at eye level while also observing attentively. These parents rarely dominated their children’s play and refrained from involvement in tasks unrelated to their children’s play. The children experienced these parents as interested and engaged in their play. We have named this first profile engaged play support.

In the second latent profile, the parents assessed themselves in various roles. These parents had medium scores on the co-player, onlooker, and uninvolved roles and low scores in the playleader role. These parents did not dominate children’s play. Compared to the first profile, however, these parents were more likely to be busy with other tasks that were not directly related to their children’s play and were less frequently co-players or observers. During their play, children experienced these parents as sometimes being very close to their play and sometimes giving them complete autonomy. We have called the second profile versatile play support.

The third profile included parents who adopt a more detached approach to their child’s play. These parents had high scores in the uninvolved role and low scores in the other three roles. They were frequently engaged in other tasks, such as housework and caring for the child’s siblings, while their child plays. This approach allowed children to experience a high level of autonomy, enabling them to play freely and independently. We have named this profile non-interfering play support.

### 3.3. Changes in Play Support over 2 Years

**Figure 2:**
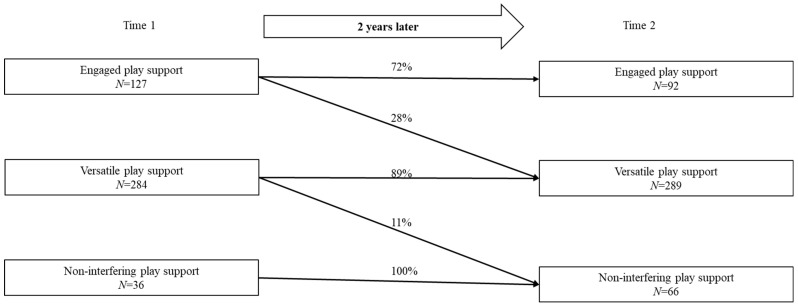
Changes in the play support profile affiliation over two years.

Figure 2 shows how the affiliation to one of the three play support profiles changed over two years. At T1, 127 mothers and fathers were classified into the engaged play support profile. Of these, 28% switched to the versatile play support profile at T2, while the remaining 72% remained in the engaged play support profile. Among the 284 mothers and fathers who were categorized in the versatile play support profile at T1, 11% switched to the non-interfering play support profile at T2 and the remaining 89% continued in the versatile play support profile. No further profile changes were observed. To summarize, these findings indicate that individual parents’ affiliations to one of the play support profiles remained relatively stable over two years.

### 3.4. Demographic Predictors of Profile Membership

A Kruskal–Wallis ANOVA was conducted to examine differences in the three profiles of parental play support in relation to child age, parental age, child gender (0 = male, 1 = female), and the presence of siblings (0 = no, 1 = yes). When significant differences were found, a pairwise comparison was calculated according to Dwass–Steel–Critchlow–Fligner’s method to see which groups showed a significant difference. As Table 5 shows, we found no differences in parental age and children’s gender, but we did find differences between the profiles according to children’s age and the presence of siblings. The engaged play support profile was used with children significantly younger than those with the other two profiles. Furthermore, parents with only one child exhibited the engaged play support profile significantly more than the versatile play support profile or the non-interfering play support profile.

### 3.5. Effects of Play Support Profiles on Children’s Playfulness

Cross-sectionally, we found no effect of any of the three profiles of parental play support on children’s playfulness (see Table 6). However, longitudinal examination of the data identified an effect: children whose parents exhibited the engaged play support profile at T1 consistently scored higher in their playfulness at T2 than did children whose parents exhibited the versatile play support profile or the non-interfering play support profile.

## 4. Discussion

This study involved 447 mothers and fathers and aimed to investigate whether different patterns in parental play support can be identified. For this purpose, latent profile analyses (LPAs) were used to divide the parents’ reported role preferences during children’s play into play support profiles. Three distinct profiles were identified that differ in their level of support. To understand whether affiliation to any of these three profiles changed over two years, latent profile transition analyses (LPTAs) were conducted. These showed that only 14% of parents changed their profile affiliation. In addition, our findings indicate that engaged play support, characterized by playing with the child at their level and attentively observing play, is positively associated with children’s playfulness longitudinally.

### 4.1. Parental Play Support Profiles

The results of the LPAs indicated that the optimal solution involved three parental play support profiles. We described these three profiles as engaged play support, versatile play support, and non-interfering play support. These three profiles showed varying degrees of parental involvement during children’s play. In the engaged play support profile, parents are highly involved and stay close to their child’s play by playing at eye level with the child or actively observing their child’s play. Around 28% of all parents were at T1 in this profile. In the versatile play support profile, parents alternate between sometimes joining in or observing their child’s play and sometimes being uninvolved to give the child autonomy and self-determination. At T1, almost 64% of all participating parents (284 mothers and fathers) belonged to this profile. This versatile play support therefore appears to be the most common form of parental play support for parents of 2 to 6-year-old children in German-speaking Switzerland. In contrast, the non-interfering play support profile is marked by parents giving their child high autonomy and independence in their play. At T1, the fewest mothers and fathers, just 8%, showed this profile.

### 4.2. Changes in the Play Support Profile Affiliation over Two Years

The LPTAs showed that profile affiliation was very stable for all three profiles over the study period of two years. Similar stability in parental play support was also evident, for example, in the longitudinal study by Hwang and Lamb (1997), who investigated the extent to which parents are involved in daily activities with their children from infancy through preschool and into elementary school. Those authors concluded that parental involvement with their children is a moderately stable behavior and that parents who changed their involvement did so because as children grow, they become more independent in their play activities and are more focused on playing with peers (Hwang & Lamb, 1997). In our study, only 65 of the 447 mothers and fathers (14.5%) changed their profile affiliation. Nevertheless, those parents who changed their affiliation did so by giving the child more autonomy and independence during their play. We did not find a single case in which the reverse was the case. This further supports the conclusion that some parents adapt their play support as the children grow.

### 4.3. Association Between Age, Gender, and Siblings and Profile Membership

Considering the age of the children and the presence of siblings, the results can be contextualized according to existing findings (Warash et al., 2017; Wustmann Seiler et al., 2021). Parents with only one child and parents with younger children were overrepresented in the engaged play support profile. Compared to parents of older children or more than one child, they were more actively involved in their child’s play. This is likely because older children develop greater independence and social skills (Takahashi et al., 2015), making them less reliant on parental support. As children grow, they engage in self-directed play, prefer peer play, and value autonomy in their play, without the need for adult support (Hwang & Lamb, 1997; Lillard et al., 2013). Additionally, siblings often serve as playmates, allowing parents to take a more passive role and withdraw from direct involvement in children’s play. Notably, the child’s gender and the parent’s age had no influence on which play support profile the parent belonged to.

### 4.4. Cross-Sectional and Longitudinal Effects of Parental Play Support on Children’s Playfulness

No effect of the play support profiles on children’s playfulness was found cross-sectionally. However, the longitudinal analyses showed that children of parents with the engaged play support profile at T1 were assessed as being more playful at T2 than were children of parents from the versatile play support and non-interfering play support profiles. This suggests that the profile in which the parents are close to children’s play and give a lot of attention to it had a positive effect on the development of children’s playfulness over time. The longitudinal effects suggest that young children’s playfulness benefits from parents who are near during play. This engaged play support may be an expression of a close relationship between parents and their children (Grossmann et al., 2002; Liang & Bi, 2025), which in turn has a positive effect on children’s playfulness (Wustmann Seiler et al., 2024).

### 4.5. Strengths and Limitations

To the best of our knowledge, this study is the first to use a person-centered approach to representing parental play support. Furthermore, for the first time, we investigated changes in profile affiliation over two years, and we examined how parental play support profiles affect children’s playfulness both cross-sectionally and longitudinally. In addition to these strengths, several limitations must be acknowledged. Firstly, to assess parental play support, we collected self-reports of their roles during children’s play without any external validation. Parental self-reports may be influenced by social desirability and potential biases, leading to overestimation or underestimation of certain parenting behaviors (Paulhus, 2017). Future studies could enhance validity by observing parents in their home environment to confirm self-reported play support profiles. Additionally, integrating the children’s perspective could also increase validity (Duss et al., 2023; Müller et al., 2015; Sturgess et al., 2002). Children could be asked what their parents are doing while they are playing and therefore, we can see whether the children see their parents as engaged, versatile, or non-interfering. Secondly, although parents reported their engagement levels in the various roles, qualitative aspects of their support were not captured. Understanding how parental play support manifests qualitatively and how this influences children’s playfulness could offer deeper insights into the dynamics of parental play support. In addition, the present study was unable to investigate whether the parents support play adaptively in relation to the children’s play and needs. This aspect could be explored through situation-specific analyses in future studies. Thirdly, we had no information on whether children actively requested parental support in their play or explicitly stated that they wanted to play independently without parental support. Further investigations into this dynamic interaction between parental support and children’s requests could help illuminate how children’s behavior influences parental play support. Fourthly, the director role had to be excluded, because otherwise the model fits were insufficient, and therefore this behavior is not part of the three profiles. However, studies have shown that directive parenting during children’s play correlates negatively with children’s playfulness (Wustmann Seiler et al., 2021); thus, it would be important to include the director role in further studies. It may be necessary to reformulate the items in a slightly weaker form to enhance their relevance and applicability to the parents. Fifthly, as the proportion of fathers was only 10%, no statements could be made as to whether the categorization into individual profiles can be explained by parents’ gender. Similarly, no conclusions can be drawn regarding whether parental gender influences potential profile changes. Further studies could benefit from including both mothers and fathers of the same children to identify differences in parental play support by parents’ gender. The same applies to the educational background of the parents and the family language and nationality. Because the participating parents tended to have a high level of education and almost all of the families spoke German and were Swiss nationals, it is not possible to make any statements about how educational and migration background affected parental play support during children’s play. Lastly, due to the recruitment strategy, all children were in a childcare center or kindergarten at T1. Within these environments, children engage in social play interactions with their peers. These peer interactions can potentially foster independence in children’s play (Casey et al., 2012; Zhao & Gibson, 2023). Consequently, children who have been exposed to such social play activities may be less dependent on their parents’ play support than children who have had few such social play experiences. Hence, the results cannot be transferred to families whose children do not attend a childcare center or kindergarten.

## 5. Conclusions

Parents of 2-to-6-year-old children support children’s play in different ways, which were grouped into three profiles. These profiles differ in how actively parents engage in their children’s play and how much autonomy they allow to their child during play. The majority of parents were in the versatile play support profile, which is characterized by a balance between parental engagement and the child’s autonomy during play. Over two years, parents tended to remain in their original play support profiles. If they did change profile, they moved to a profile in which they are less involved in their child’s play and where the child has more autonomy in their play. These changes are presumably a function of children growing and becoming more independent. Furthermore, there is evidence of a positive longitudinal effect of the engaged play support profile on children’s playfulness. Playing with the child at eye level and observing the child’s play attentively seems to have a positive effect on children’s playfulness. This type of play support communicates to the children that their play is seen and valued as important. Additionally, such engaged play support reflects a strong parent–child relationship, which further enhances children’s playfulness (Wustmann Seiler et al., 2024).

## Figures and Tables

**Table 1:** Demographic characteristics at T1.

	*N* = 447
Children’s gender (female/male)	208/239
Children’s age in months (SD)	58.2 (16.9)
*Number of siblings*	
No siblings	86 (18.3%)
One sibling	262 (55.9%)
Two or more siblings	99 (21.1%)
*Educational setting of the child*	
Childcare center	197 (44%)
Kindergarten	250 (56%)
*Nationality*	
Swiss	400 (89%)
Other	47 (11%)
*Family language*	
German or Swiss-German	422 (94%)
Other	25 (6%)
*Mothers highest Education (N = 401)*	
Vocational education	138 (34%)
Academic	247 (62%)
Missing	16 (4%)
*Fathers highest Education (N = 46)*	
Vocational education	11 (24%)
Academic	35 (76%)
Missing	0 (0%)

**Table 2:** Descriptive statistics and intercorrelations between all study variables.

		*N*	*M*	*SD*	1	2	3	4	5	6	7	8	9	10	11	12	13
1	Co-Player T1	447	3.83	0.74	-												
2	Playleader T1	447	2.70	0.73	0.04	-											
3	Onlooker T1	447	3.48	0.63	0.32 ***	0.22 ***	-										
4	Uninvolved T1	447	3.73	0.71	−0.15 **	−0.14 **	−0.26 ***	-									
5	Co-Player T2	447	3.51	0.79	0.59 ***	0.03	0.27 ***	−0.19 ***	-								
6	Playleader T2	447	2.78	0.67	0.08	0.51 ***	0.25 ***	−0.16 ***	0.16 ***	-							
7	Onlooker T2	447	3.27	0.64	0.29 **	0.15 **	0.57 ***	−0.20 ***	0.33 ***	0.34 ***	-						
8	Uninvolved T2	447	3.84	0.66	−0.14 **	−0.12 *	−0.17 ***	0.54 ***	−0.22 ***	−0.19 ***	−0.21 ***	-					
9	Age (in months)	447	58.18	16.86	−0.23 ***	−0.16 ***	−0.21 ***	0.17 ***	−0.25 ***	−0.20 ***	−0.17 ***	0.16 ***	-				
10	Gender (female)	447	0.46	-	0.00	−0.01	−0.02	0.08	0.03	0.00	−0.07	−09	−0.04	-			
11	Children’s playfulness T1	446	3.92	0.41	−0.04	−0.14 **	−0.00	0.11 *	−0.02	−0.05	−0.02	0.09 *	0.32 ***	0.04	-		
12	Children’s playfulness T2	446	4.00	0.39	0.08	−0.06	0.12 **	−0.01	0.14 **	0.04	0.21 ***	0.02	−0.09 *	0.02	0.44 ***	-	
13	Siblings (yes)	443	0.84	-	−0.17 ***	−0.08	−0.20 ***	0.13 **	−0.17 ***	−0.15 **	−0.10 *	0.19 ***	0.22 ***	−0.05	0.15 **	0.04	-
14	Parental age	446	39.11	4.53	−0.14 **	−0.06	−0.10 *	−0.07	−0.04	−0.12 *	−0.11 *	−0.09	0.22 ***	0.04	−0.06	−0.04	0.01

*Note. M* = mean; *SD* = standard deviation; * *p* < 0.05; ** *p* < 0.01; *** *p* < 0.001.

**Table 3:** Model fit indices for latent profile analysis of parental play support.

	AIC	BIC	SABIC	Entropy	ALMR LR Test *p*-Value	Classification Accuracy	Group Size	BLRT *p*-Value
*T1*								
1-Profile	3818.58	3851.40	3826.01	-	-	-	100%	-
2-Profiles	3756.28	3809.61	3768.35	0.393	*p* = 0.359	0.792–0.802	51.2–48.6%	*p* = 0.000
3-Profiles	3708.50	3782.35	3725.22	0.748	*p* = 0.004	0.846–0.901	6.2–71.4%	*p* = 0.000
4-Profiles	3696.44	3790.80	3717.81	0.810	*p* = 0.094	0.856–0.902	1.6–70.9%	*p* = 0.000
5-Profiles	3680.60	3795.47	3706.61	0.844	*p* = 0.000	0.838–0.999	0.4–68.7%	*p* = 0.000
6-Profiles	3671.56	3806.95	3702.22	0.855	*p* = 0.068	0.838–0.999	0.1–68.5%	*p* = 0.040
*T2*								
1-Profile	3750.23	3783.05	3757.66	-	-	-	100%	-
2-Profiles	3637.89	3691.22	3649.97	0.539	*p* = 0.000	0.832–0.875	36.2–63.8%	*p* = 0.000
3-Profiles	3616.31	3690.16	3633.03	0.692	*p* = 0.061	0.821–0.866	4.4–60.2%	*p* = 0.000
4-Profiles	3581.19	3675.55	3602.56	0.780	*p* = 0.294	0.792–0.893	3.6–47.7%	*p* = 0.000
5-Profiles	3570.04	3684.91	3596.05	0.809	*p* = 0.124	0.797–0.904	1.8–47.7%	*p* = 0.000
6-Profiles	3564.22	3699.60	3594.87	0.814	*p* = 0.279	0.768–0.969	0.1–47.4%	*p* = 0.088

*Note.* AIC = Akaike information criterion; BIC = Bayesian information criterion; SABIC = sample-size adjusted BIC; ALMR LR = Adjusted Lo–Mendell–Rubin Likelihood Ratio Test; BLRT = bootstrap likelihood ratio test.

**Table 4:** Single-factor ANOVA to test for differences in individual roles across profiles.

	Profile Affiliation			F-Ratio	Sig. Level	Games–Howell Post Hoc Test (Difference in Mean)		
	Engaged Play Support *M (SD)*	Versatile Play Support *M (SD)*	Non-Interfering Play Support *M (SD)*			1 and 2	1 and 3	2 and 3
Co-Player T1	4.31 (0.56)	3.70 (0.66)	3.15 (0.94)	57.3	<0.001	−0.61 ***	−1.15 ***	−0.55 **
Playleader T1	3.00 (0.76)	2.61 (0.67)	2.34 (0.68)	16.9	<0.001	−0.39 ***	−0.66 ***	−0.27
Onlooker T1	4.17 (0.37)	3.33 (0.36)	2.28 (0.38)	430.7	<0.001	−0.84 ***	−1.89 ***	−1.05 ***
Uninvolved T1	3.39 (0.75)	3.80 (0.63)	4.39 (0.56)	39.0	<0.001	0.42 ***	1.00 ***	0.59 ***

*Note.* 1 = Engaged play support; 2 = Versatile play support; 3 = Non-interfering play support; *M* = mean; *SD* = standard deviation; ** *p* < 0.01; *** *p* < 0.001.

**Table 5:** Kruskal–Wallis ANOVA to test for differences between the Profiles according to children’s age and gender and the presence of siblings.

	χ^2^	*df*	*p*	Dwass–Steel–Critchlow–Fligner (Difference in W)		
				1 and 2	1 and 3	2 and 3
Children’s age	31.752	2	<0.001	7.12 ***	5.82 ***	2.51
Parental age	3.550	2	0.170	-	-	-
Gender	0.197	2	0.908	-	-	-
Presence of siblings	40.441	2	<0.001	8.17 ***	5.21 ***	2.06

*Note.* 1 = Engaged play support; 2 = Versatile play support; 3 = Non-interfering play support; χ^2^ = Chi-Quadra; *df* = degree of freedom; W = rank sums; *** *p* < 0.001.

**Table 6:** Linear regression analysis predicting children’s playfulness at both measurements.^1^

	Playfulness T1 *β* (SE)	Playfulness T2 *β* (SE)
Intercept	3.46 (0.07) ***	2.42 (0.16) ***
Playfulness T1	-	0.50 (0.04) ***
Non-interfering play support	−0.09 (0.08)	−0.13 (0.07) *
Versatile play support	−0.07 (0.04)	−0.07 (0.04) ^†^
Age (in months)	0.01 (0.00) ***	−0.01 (0.00) ***
Gender	0.04 (0.04)	−0.01 (0.03)
R^2^	0.11	0.27

*Note.* Engaged play support was used as reference category; R^2^ = determination coefficient; *β* = beta coefficient; SE = standard error; ^†^ *p* < 0.10, * *p* < 0.05, *** *p* < 0.001.

## Data Availability

The data presented in this study are available on request from the corresponding author due to the upcoming public release of the complete project data.

## Note

1 When children’s playfulness was assessed solely through parent ratings, the cross-sectional associations with parental play support profiles were significant, whereas no significant associations were found when using only teacher ratings (see Appendix C for full results).

## Appendix A

**Table A1:** Items of the questionnaire on parental play support roles in English and German.

	German Version	English Version
	**Co-Player**	
1.	Wenn mein Kind möchte, dass ich mitspiele, passe ich mich seinem Spielfluss an und überlasse ihm die Führung des Spiels.	When my child invites me to play with him/her, I go with his/her flow and let him/her direct the play.
2.	Wenn mein Kind möchte, dass ich mitspiele, beteilige ich mich als gleichberechtigte/r Spielpartner/in (indem ich mich ohne Absichten auf sein Spiel einlasse).	When my child invites me to play with him/her, I participate as an equal (by joining in his/her play without pursuing objectives).
3.	Wenn mein Kind möchte, dass ich mitspiele, lasse ich mich auf seine Spielideen ein (z.B. indem ich mich zu ihm auf den Boden setze oder Rollen übernehme, die es mir zuteilt).	When my child invites me to play with him/her, I go along with his/her play ideas (for example, by sitting down on the floor with him/her or playing a role that he/she gives me).
	**Playleader**	
4.	Wenn mein Kind nicht ins Spiel findet, unterstütze ich es mit Spielvorschlägen.	If my child does not engage in play, I encourage him/her by making play suggestions.
5.	Wenn ich merke, dass sich das Spiel meines Kindes erschöpft, schlage ich ihm andere Spielmöglichkeiten vor.	When my child loses interest in what he/she is playing, I suggest other things to play.
6.	Wenn ich merke, dass das Spiel meines Kindes ins Stocken gerät, gebe ich ihm Hinweise, wie es sein Spiel erweitern kann.	When I notice my child’s play is about to wind down, I give suggestions on how he/she can extend his/her play.
	**Onlooker**	
7.	Ich schaue meinem Kind während seines Spiels aufmerksam zu.	I watch my child with attention when he/she plays.
8.	Ich beobachte das Spiel meines Kindes, um Erkenntnisse über seine aktuellen Themen und Interessen zu gewinnen.	I observe my child’s play to learn about his/her current interests and favorite topics.
9.	Ich bin während des Spiels präsent und in der Nähe meines Kindes.	While my child is playing, I am present and nearby.
	**Uninvolved**	
10.	Während mein Kind spielt, erledige ich organisatorische, administrative Aufgaben (z.B. Telefonate, Einkaufslisten, Papierkram).	When my child plays, I do organizational, administrative tasks (such as make telephone calls, make grocery list, do paperwork).
11.	Während mein Kind spielt, erledige ich Haushaltstätigkeiten (z.B. Putzen, Waschen, Gartenarbeit).	While my child is playing, I do housework (such as cleaning, laundry, work in garden).
12.	Während mein Kind spielt, bereite ich Mahlzeiten zu.	While my child is playing, I cook meals.

*Note:* Items written in italics were excluded from the final analyses; Likert scale: Very rarely (1), Rarely (2), Occasionally (3), Often (4), and Very often (5).

## Appendix B

**Table A2:** Model fit comparison of the test for measurement invariance for the roles of parental play support.

	*χ* ^2^	*df*	*p*	CFI	RMSEA	SRMR	∆*χ*^2^	∆*df*	*p*	∆CFI
Configural	290.393	212	0.000	0.980	0.024	0.041				
Metric	296.486	220	0.000	0.980	0.023	0.042	6.505	8	0.591	0.000
Scalar	359.545	228	0.000	0.966	0.030	0.045	64.826	8	0.000	−0.014
Partial scalar	301.099	224	0.000	0.980	0.023	0.042	4.522	4	0.340	0.000

*Note. χ*^2^ = chi-square; *df* = degrees of freedom; *p* = probability of type I error; CFI = Comparative Fit Index; RMSEA = Root Mean Square Error of Approximation; SRMR = Standardized Root Mean Square Residual; ∆ = Difference Value; ∆*χ*^2^ = after Satorra–Bentler. Partial Scalar (4 items were excluded).

## Appendix C

**Table A3:** Linear regression analysis predicting children’s playfulness at both measurements based on parent-rated playfulness.

	Playfulness T1 *β* (SE)	Playfulness T2 *β* (SE)
Intercept	3.81 (0.07) ***	1.96 (0.15) ***
Playfulness T1	-	0.60 (0.04) ***
Non-interfering play support	−0.23 (0.08) **	−0.07 (0.06)
Versatile play support	−0.14 (0.04) **	−0.07 (0.03) ^†^
Age (in months)	0.01 (0.00) ***	−0.00 (0.00) ***
Gender	0.03 (0.04)	−0.01 (0.03)
R^2^	0.07	0.40

*Note.* Engaged play support was used as reference category; R^2^ = determination coefficient; *β* = beta coefficient; SE = standard error; ^†^ *p* < 0.10, ** *p* < 0.01, *** *p* < 0.001.

**Table A4:** Linear regression analysis predicting children’s playfulness at both measurements based on teacher-rated playfulness.

	Playfulness T1 *β* (SE)	Playfulness T2 *β* (SE)
Intercept	2.99 (0.11) ***	3.13 (0.21) ***
Playfulness T1	-	0.27 (0.06) ***
Non-interfering play support	0.12 (0.11)	−0.15 (0.13)
Versatile play support	0.03 (0.06)	−0.04 (0.08)
Age (in months)	0.01 (0.00) ***	−0.01 (0.00) **
Gender	0.07 (0.06)	0.00 (0.07)
R^2^	0.13	0.10

*Note.* Engaged play support was used as reference category; R^2^ = determination coefficient; *β* = beta coefficient; SE = standard error; ** *p* < 0.01, *** *p* < 0.001.
